# Chromatin remodeling gene *ARID2* targets cyclin D1 and cyclin E1 to suppress hepatoma cell progression

**DOI:** 10.18632/oncotarget.10244

**Published:** 2016-06-23

**Authors:** Yujie Duan, Ling Tian, Qingzhu Gao, Li Liang, Wenlu Zhang, Yi Yang, Yaqiu Zheng, E Pan, Shengwei Li, Ni Tang

**Affiliations:** ^1^ Key Laboratory of Molecular Biology for Infectious Diseases (Ministry of Education), Institute for Viral Hepatitis, Department of Infectious Diseases, The Second Affiliated Hospital, Chongqing Medical University, Chongqing, PR China

**Keywords:** ARID2, SWI/SNF complex, the Rb/E2F pathway, hepatocellular carcinoma, cell-cycle arrest

## Abstract

Exome and whole-genome sequencing studies have drawn attention to the role of somatic mutations in SWI/SNF chromatin remodeling complexes in the carcinogenesis of hepatocellular carcinoma (HCC). Here, we explored the molecular mechanisms underlying the biological roles of AT-rich interactive domain 2 (ARID2) in the pathogenesis of HCC. We found that *ARID2* expression was significantly downregulated in HCC tissues compared with non-tumorous tissues. Restoration of *ARID2* expression in hepatoma cells was sufficient to suppress cell proliferation and tumor growth in mice, whereas *ARID2* knockdown contributed to the enhancement of cellular proliferation and tumorigenicity. Suppression of *ARID2* expression accelerated G1/S transition associated with upregulation of cyclin D1, cyclin E1, CDK4, and phosphorylation of the retinoblastoma protein (Rb). Furthermore, we demonstrated that ARID2 physically interacts with E2F1 and decreases binding of E2F1/RNA Pol II to the promoters of *CCND1* and *CCNE1*. Taken together, these results demonstrate that ARID2 suppresses tumor cell growth through repression of cyclin D1 and cyclin E1 expression, thereby retarding cell cycle progression and cell proliferation in hepatoma cells. These findings highlight the potential role of ARID2 as a tumor growth suppressor in HCC.

## INTRODUCTION

Hepatocellular carcinoma (HCC) is the third leading cause of cancer-related deaths worldwide. Numerous genetic, epigenetic, and environment factors are involved in tumorigenesis, growth, and metastasis in this disease. Recent exome and genome-wide sequencing studies have provided extensive insights into the mechanisms underlying the genetic basis of HCC. However, the biological functions of currently identified candidate genes are still largely unknown.

The mammalian SWI/SNF (mating type switch/sucrose non-fermenting) complexes mediate ATP-dependent chromatin remodeling processes that are critical to the modulation of gene expression in diverse cellular processes such as cell differentiation and proliferation [1]. Loss of SWI/SNF function has been associated with malignant transformation, and recent data demonstrate that several components of the SWI/SNF complexes, including ARID1A, ARID2, SMARCA4 (BRG1), SMARCB1 (SNF5), and PBRM1, function as tumor suppressors [2–4].

ARID family proteins are characterized by the presence of a consensus N-terminal AT-rich DNA interaction domain (ARID), a RFX-type winged-helix, a proline- and glutamine-rich domain, and two conservative C-terminal C2H2 zinc finger motifs [5]. The ARID proteins participate in various central biological processes such as cell cycle control, transcriptional regulation, DNA damage response, cell lineage differentiation, and chromatin remodeling [6–10].

Recently, *ARID2* has been identified as a novel tumor suppressor gene. Frequent inactivating mutations in this gene were first observed in HCC (6.5%) [11,12], followed by melanoma (7%) [13], non-small lung carcinoma (5%) [14], and colorectal cancer (13%) [15]. Inactivating mutations have been shown to comprise missense, frameshift, and nonsense mutations distributed along the entire coding region of the *ARID2* gene. Among these, nonsense mutations in the ARID motif have been reported to potentially disrupt the DNA-binding capacity of the ARID2 protein [15]. However, the mechanism regulating ARID2 expression and function in HCC remains unknown.

In this study, we found that ARID2 expression is significantly downregulated in HCC tissues compared with adjacent nontumoral liver tissues. We additionally investigated the roles of ARID2 in the suppression of cellular proliferation and tumor growth in hepatoma cell lines. Our data suggest that ARID2 inhibits hepatoma cell-cycle progression and tumor growth by targeting the Rb-E2F signaling pathway.

## RESULTS

### ARID2 deficiency is prevalent in human hepatocellular carcinoma

**Figure 1:**
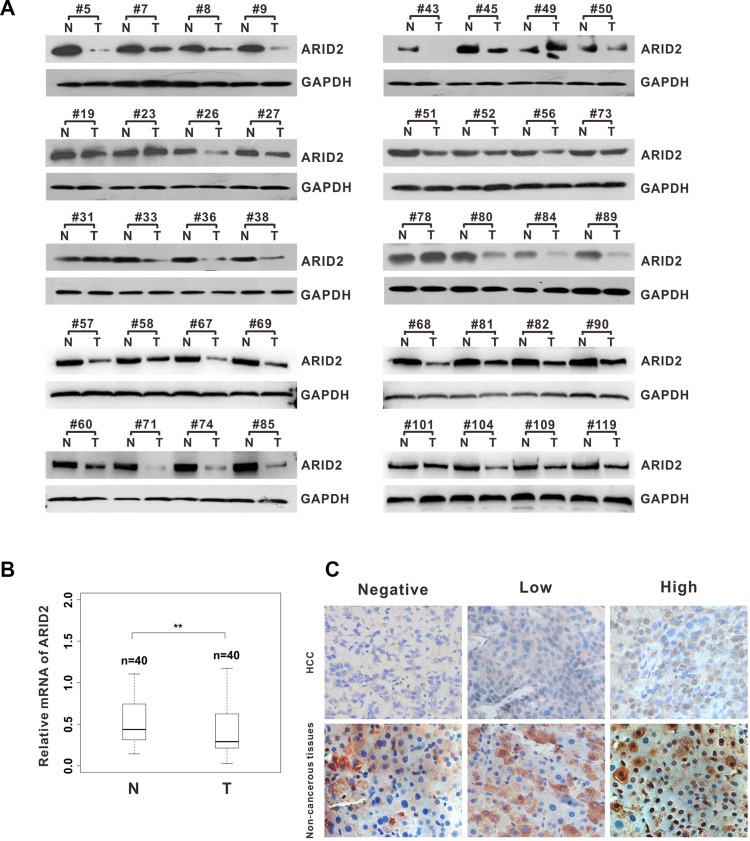
*ARID2* expression is downregulated in human hepatocellular carcinoma tissues (**A**) Western blot analysis of ARID2 expression in hepatocellular carcinoma (HCC) tissues and adjacent non-tumorous tissues (T/N). Equal loading was confirmed using GAPDH as a loading control. (**B**) Box plots of ARID2 mRNA expression in 40 paired HCC tissues; **p < 0.01 (**C**) Immunohistochemical staining of ARID2 in HCC tissues and adjacent non-tumorous tissues; magnification: 400×.

In order to investigate the potential role of *ARID2* in HCC development, we first examined the expression pattern of ARID2 in paired HCC tissues from 40 patients. Data revealed that the levels of both ARID2 transcripts and proteins were markedly lower in the tumor tissues but much higher in the peritumoral liver tissues, as shown by both RT-PCR and western blot analysis (Figure 1A and 1B). Next, we analyzed ARID2 expression in 40 paired-HCC tissues and adjacent nontumoral liver tissues by immunohistochemistry (IHC) staining. The IHC score of nuclear immunoreactivity to ARID2 were classified as negative (score 0), low (score 1–2) and high (score 3) (Figure 1C). Correlative analysis of ARID2 protein levels with clinicopathologic features suggested that lower expression of ARID2 protein was closely associated with poor tumor differentiation (*p* < 0.01; Supplementary Table 1). However, no significant correlation was found between ARID2 expression and other clinicopathological parameters such as age, gender, tumor size, or metastasis (Supplementary Table 1). These data suggest that ARID2 plays a clinically relevant role as a tumor growth suppressor in HCC.

### Suppression of *ARID2* promotes cell proliferation by inducing G1/S transition in hepatoma cells

**Figure 2:**
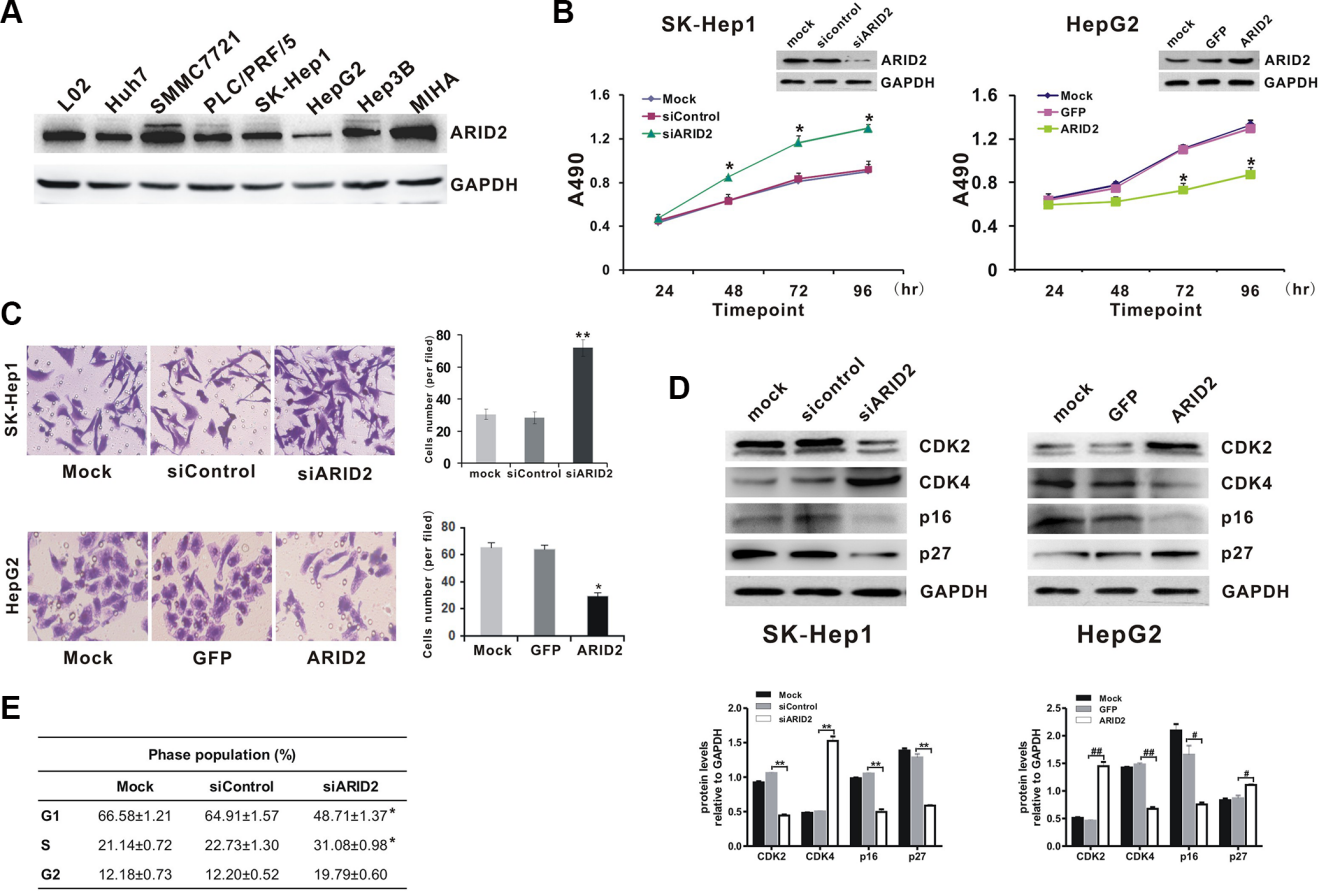
Suppression of *ARID2* expression promotes cell proliferation by inducing G1/S transition in hepatoma cells (**A**) Endogenous expression levels of ARID2 protein in hepatoma cell lines LO2, Huh7, SMMC-7721, PLC/PRF/5, SK-Hep1, HepG2, Hep3B, and MIHA (**B**) Cell proliferation curves. SK-Hep1 cells were infected with adenoviruses expressing siRNA targeting ARID2 (AdR-siARID2) or siRNA control (AdR-siControl). HepG2 cells were infected with adenoviruses expressing ARID2 (Ad-ARID2) or vector control (Ad-GFP). At 12 hours after infection, cells were plated into 24-well plates at 0.5 × 10^4^ cells/ml and counted every 24 hours in triplicate. Data are presented as means ± SD; *p < 0.05 vs. control. (**C**) Transwell assay of cell migration in SK-Hep1 or HepG2 cells. Data represent the results of three independent experiments ± SD; *p < 0.05; **p < 0.01 vs. vector control; magnification: 200 × (**D**) and (**E**) Cell-cycle analysis and detection of cell cycle proteins. Sk-Hep1 and HepG2 cells were treated as mentioned in Figure 2B. Cell lysates were subjected to western blot analysis for CDK2, CDK4, p16, and p27. Results shown are representative samples from at least three independent experiments. Integrated density of these cell cycle proteins was quantitatively analyzed using ImageJ software; *p < 0.05, **p < 0.01 (siARID2 vs. siControl); ^#^*p* < 0.05, ^##^*p* < 0.01 (ARID2 vs. GFP) (E) At 96 hours post-infection, cells were collected and subjected to flow cytometry. Populations of cells at G1, S, and G2/M phases are indicated as percentages of the whole cell population. Data represent the results of three independent experiments ± SD;*p < 0.05 vs. vector control.

We next evaluated the effect of ARID2 on cell proliferation using the hepatoma cell lines SK-Hep1, HepG2, and SMMC-7721. Results indicated strong endogenous *ARID2* expression in LO2, MIHA, and SMMC-7721 cells, modest expression in SK-Hep1 cells, PLC/PRF/5, and Hep3B cells, and low expression levels in HepG2 and Huh7 cells (Figure 2A). Then, we constructed *ARID2*-overexpression or *ARID2*-knockdown vectors according to the endogenous expression patterns in hepatoma cells. We found that overexpression of *ARID2* significantly suppressed cell proliferation and migration in both HepG2 cells and SMMC-7721 cells (Figure 2B, 2C, and Supplementary Figure 1A). *ARID2* silencing increased proliferation rates and enhanced migration capacity in SK-Hep1 cells and SMMC-7721 cells (Figure 2B, 2C, and Supplementary Figure 1A). However, the vector or scrambled siRNA control had no effect on cell proliferation, indicating that the effect elicited by *ARID2* was highly specific.

In order to determine whether the impact of ARID2 on cell proliferation is related to cell-cycle control, we analyzed the effect of ARID2 on the cell cycle by flow cytometry. The results indicated that *ARID2* knockdown significantly promoted G1- to S-phase transition in SK-Hep1 cells, especially at 96 hours post-infection (Figure 2E). Taken together, the above data strongly suggest that suppression of *ARID2* expression promotes cell proliferation and facilitates G1/S transition in hepatoma cells.

Next, we compared the expression levels of cell cycle-related proteins by western blotting. The data showed that the ectopic expression of *ARID2* induced marked upregulation of CDK2 and p27 proteins in HepG2 cells, whereas p16 and CDK4 exhibited sharply decreased expression in *ARID2*-expressing cells. In contrast, depletion of ARID2 resulted in the upregulation of CDK4 expression in SK-Hep1 cells, whereas the expression of p16, p27, and CDK2 was significantly downregulated (Figure 2D). Similar results were obtained in SMMC-7721 cells (Supplementary Figure 2E). These data indicate that knockdown of *ARID2* promotes the progression of cells into S phase.

### ARID2 controls the expression of Rb-E2F-dependent genes

**Figure 3:**
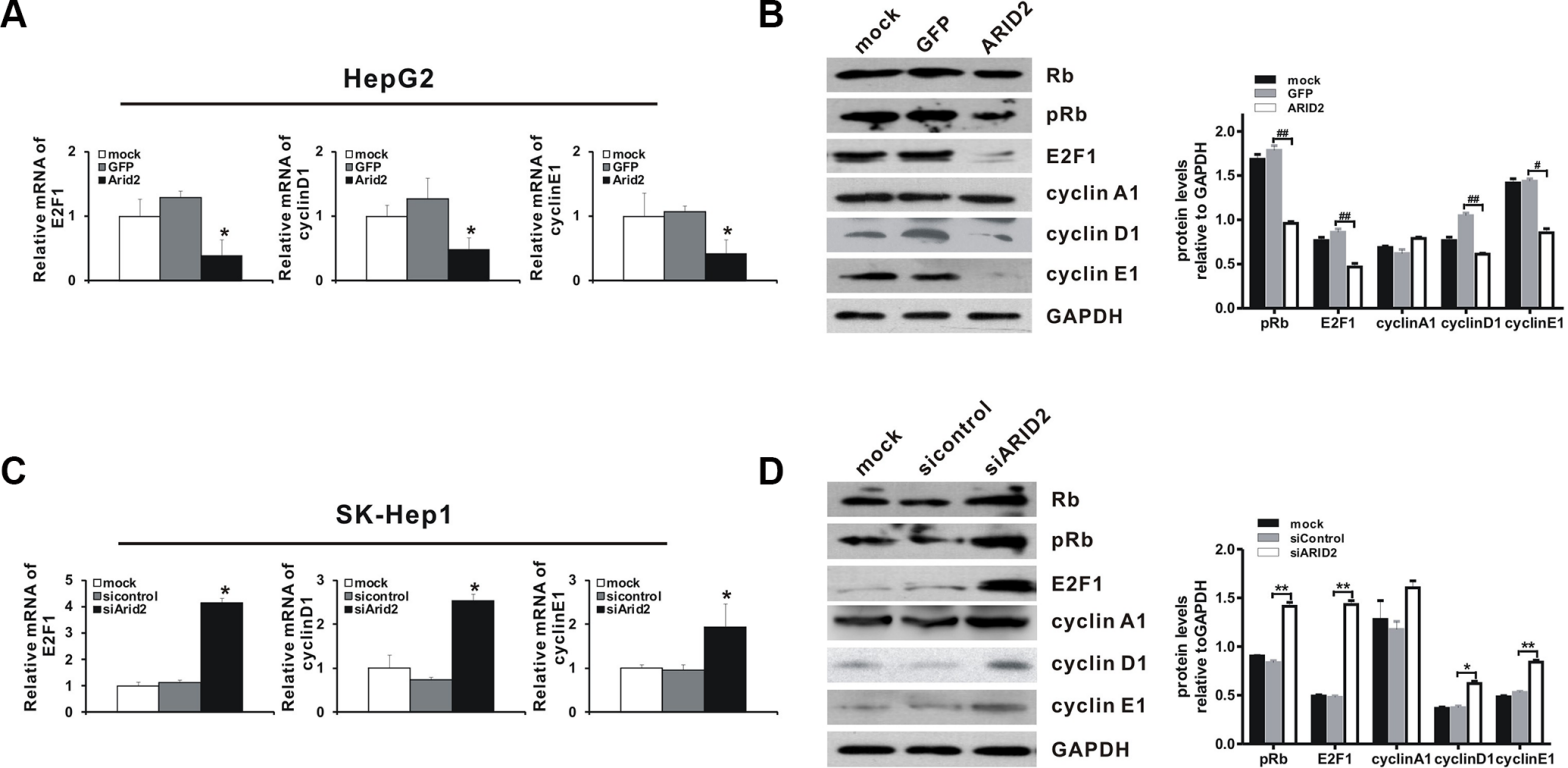
ARID2 controls the expression of Rb-E2F-dependent genes SK-Hep1 cells and HepG2 cells were treated as described in Figure 2B. At 24 hours and 36 hours after infection, total RNA and whole protein lysates were extracted, respectively. The RNA and protein levels of selected Rb-E2F-dependent genes, including those encoding Rb, phosphorylated-Rb (p-Rb), E2F1, cyclin D1, cyclin E1, and cyclin A, were determined by RT-qPCR (**A** and **C**) and western blotting (**B** and **D**). Data represent the means ± SD; *p < 0.05 vs. vector control. Immunoblot density was quantitatively analyzed using ImageJ software; *p < 0.05, **p < 0.01 (siARID2 vs. siControl); ^#^*p* < 0.05, ^##^*p* < 0.01(ARID2 vs. GFP).

Then, we investigated the mechanisms by which ARID2 affects the cell cycle-dependent expression of a subset of Rb-E2F target genes. Our results showed that ectopic expression of *ARID2* decreased the expression of E2F1 and phosphorylated-Rb (pRb) in both HepG2 and SMMC-7721 cells (Figure 3A, 3B, Supplementary Figure 1B, and Supplementary Figure 2A, 2B, 2D). Cyclins are groups of proteins that regulate the progression through the cell cycle by activating cyclin-dependent kinases (CDKs). We additionally examined the levels of various cyclins and other cell cycle-activating proteins in *ARID2*-expressing HepG2 and SMMC-7721 cells. Our results demonstrate that ARID2 decreases the levels of cyclin D1 and cyclin E; however, the level of cyclin A was not affected (Figure 3A, 3B, Supplementary Figure 1C, and Supplementary Figure 2B, 2C, 2D).

We next explored the effects of ARID2 depletion on the expression of Rb-E2F targets. As expected, *ARID2* knockdown strongly increased the expression of pRb, E2F1, and downstream targets cyclin D1 and cyclin E1 in both Sk-Hep1 and SMMC-7721 cells (Figure 3C, 3D, Supplementary Figure 1D, 1E, and Supplementary Figure 2). The expression of cyclin A1 was slightly increased after ARID2 depletion in these cells (Figure 3D and Supplementary Figure 2D). Taken together, these data demonstrate that ARID2 plays an important role in controlling the expression of Rb-E2F-target genes.

### ARID2 inhibits hepatoma growth in orthotopic and xenograft implantation models

As ARID2 induces cyclin D1 and cyclin E1 repression, thereby inhibiting the proliferation of hepatoma cells, this protein may be critical for the suppression of HCC tumorigenesis *in vivo*. In order to test this hypothesis, we examined the effects of knockdown or restoration of ARID2 on the growth of hepatoma cells in murine xenograft and orthotopic models.

**Figure 4:**
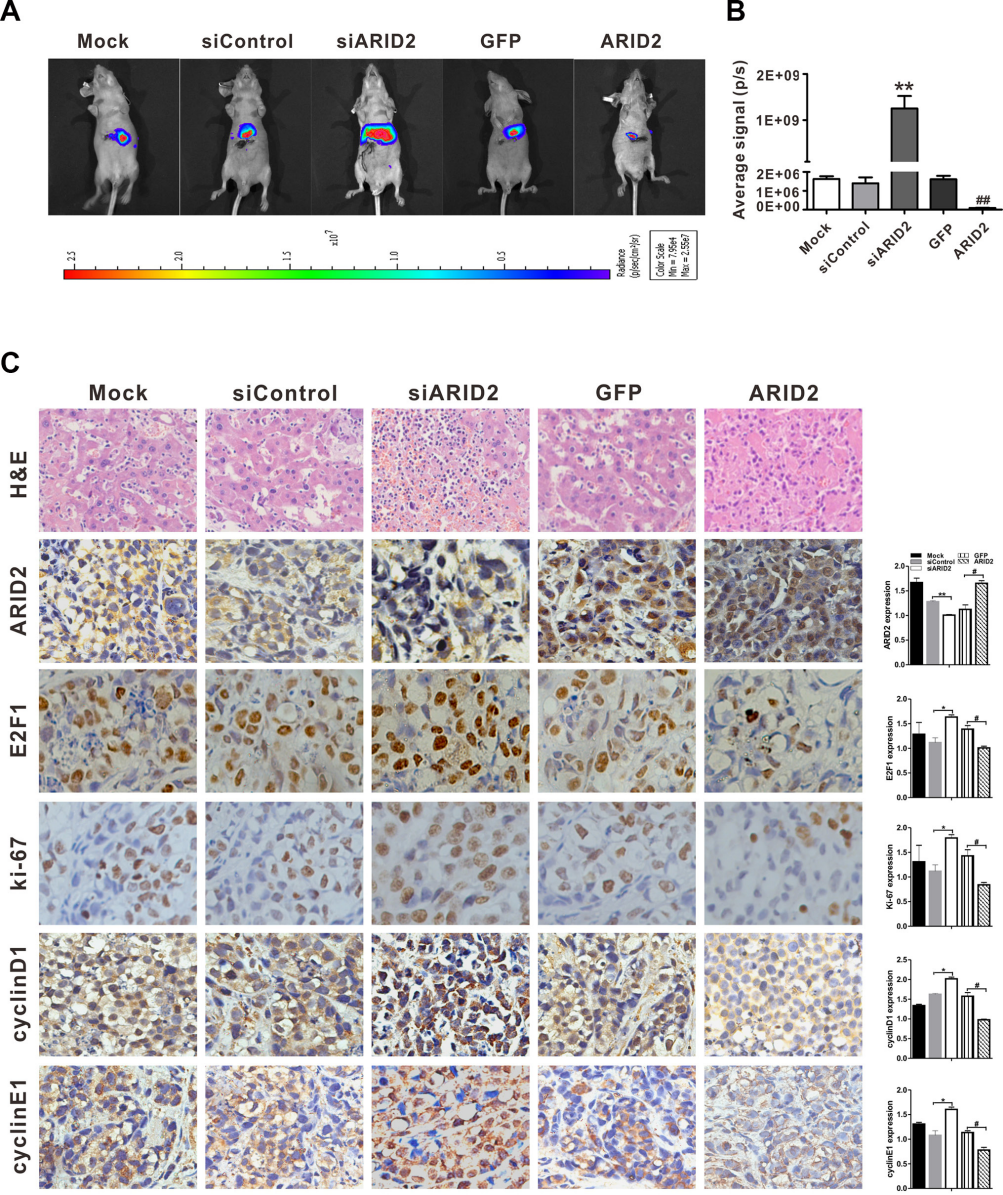
ARID2 inhibits hepatoma growth in the orthotopic implantation model SMMC-7721 cells were co-infected with AdR-FLuc and AdR-siARID2, AdR-siControl, Ad-GFP, or Ad-ARID2 for 24 hours. The infected cells (1 × 10^5^ cells/injection) were implanted into the left lobes of the livers of nude mice (5 mice per group). (**A**) Representative bioluminescent imaging (BLI) at 7 weeks following orthotopic implantation; the colored scale bar represents bioluminescence intensity (**B**) Xenogen BLI analysis; the Xenogen imaging signal intensity was quantitatively analyzed; **p < 0.01 (siARID2 vs. siControl); ^##^*p* < 0.01 (ARID2 vs. GFP) (**C**) Representative H&E staining or immunohistochemical detection of ARID2, E2F1, ki-67, cyclin D1, and cyclin E1 in liver tumor tissues; magnification: 400 ×. Immunostaining intensity was analyzed using Image-Pro plus 6.0 software; *p < 0.05, **p < 0.01(siARID2 vs. siControl); ^#^*p* < 0.05, ^##^*p* < 0.01 (ARID2 vs. GFP).

SMMC-7721 cells were infected with Ad-ARID2, Ad-GFP, AdR-siARID2, or AdR-siControl, labeled with RFP-luciferase, and subsequently transplanted into the livers of nude mice. As shown in Figure 4A and 4B, significant repression of tumor growth was observed to occur in nude mice following restoration of *ARID2* expression. However, knockdown of *ARID2* significantly promoted tumor formation in orthotopic tumor models. The xenograft model evidenced similar tumor-retarding effects of ARID2 (Supplementary Figure 3A).

As *ARID2* repression is closely correlated with cell proliferation and invasiveness, the expression of cell growth and aggressiveness markers Ki-67, E2F1, cyclin D1, and cyclin E1 were then evaluated in tumor tissues. Histological analysis further confirmed that the expression of Ki-67, E2F1, cyclin D1, and cyclin E1 was remarkably decreased in tumor tissues following the exogenous expression of *ARID2*. In contrast, *ARID2* knockdown markedly enhanced the expression of these markers (Figure 4C). These findings were consistent with those in xenograft tumor models (Supplementary Figure 3B). Taken together, these results imply that restoration of *ARID2* expression inhibited tumor growth and aggressiveness *in vitro* and *in vivo*.

### ARID2 represses binding of E2F1 to the *CCND1* and *CCNE1* promoter regions to silence cyclin D1 and cyclin E1 expression

**Figure 5:**
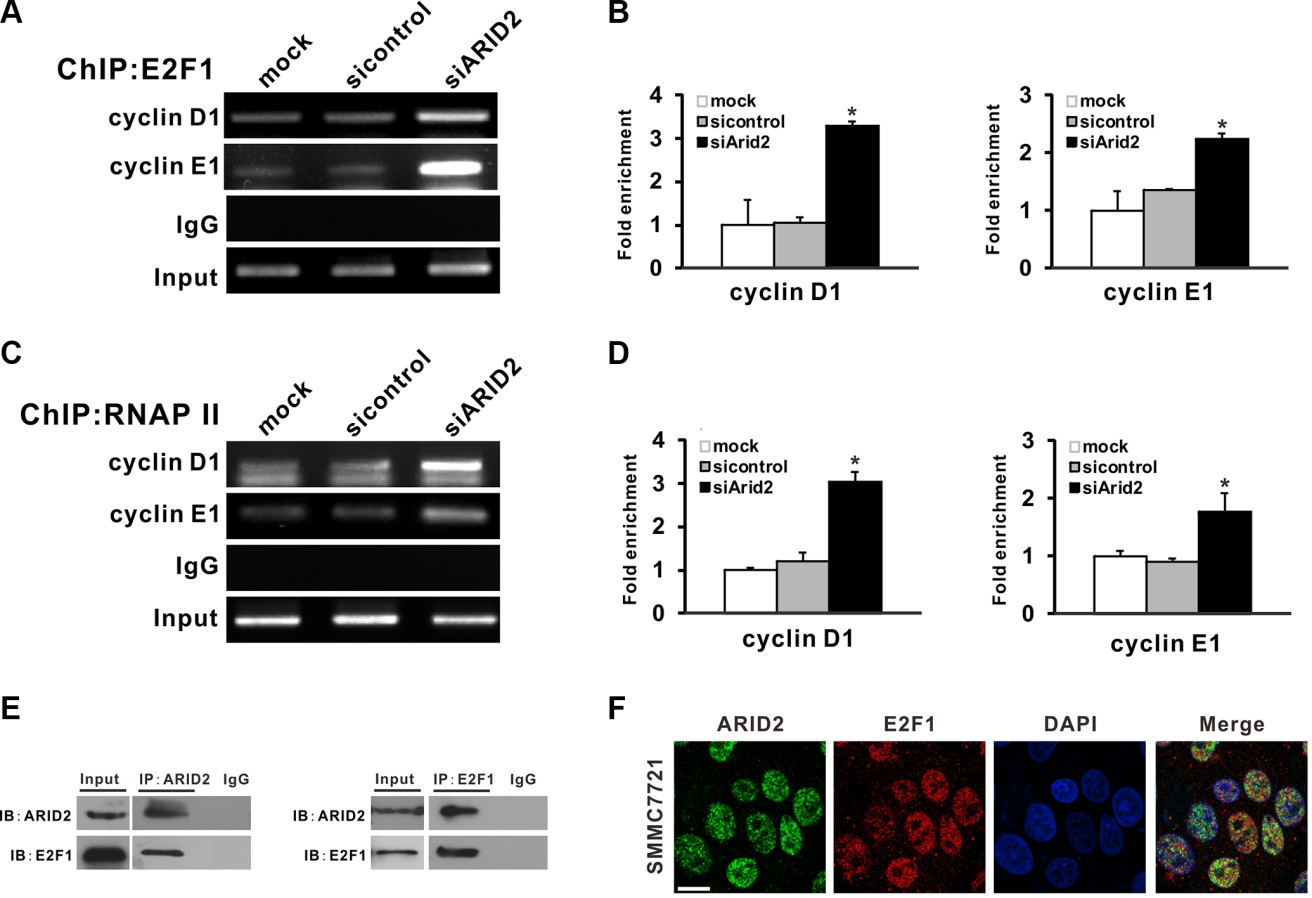
ARID2 interacts with E2F1 and reduces E2F1 binding with *CCND1* and *CCNE1* promoters (**A–D**) SK-Hep1 cells were infected with control siRNA (AdR-siControl) or siRNA against ARID2 (AdR-siARID2). After 24 hours, cell lysates were collected to perform ChIP analysis of E2F1 (A) or RNA Pol II (C) on the *CCND1* (cyclin D1) and *CCNE1* (cyclin E1) promoters. IgG served as negative control. The relative fold-enrichment (bound/input) was measured by qPCR (B and D). Data represent the means of three independent experiments ± SD; *p < 0.05 vs. siControl. (**E**) Physical interaction between endogenous E2F1 and ARID2. Cell extracts were immunoprecipitated separately with anti-ARID2 or anti-E2F1 antibodies, and the associated E2F1 and ARID2 were examined respectively by western blotting. (**F**) Nuclear colocalization of ARID2 and E2F1. Immunofluorescence staining of SMMC-7721 cells using monoclonal anti-ARID2 (green) and anti-E2F1(red) antibodies. DAPI (blue) was used to counterstain nuclei. Representative images are shown; scale bar: 30 μm.

Our results indicated that ARID2 is essential for the inhibition of cell proliferation and suppression of E2F-dependent gene expression. Therefore, we investigated the molecular mechanisms by which ARID2 represses cyclin D1 and cyclin E1 in SMMC-7721 and SK-Hep1 cells. Chromatin immunoprecipitation (ChIP) assays revealed that the overexpression of *ARID2* impaired binding of E2F1, RNA polymerase II, and acetylated histone modification (AcH3 and AcH4) at the *CCND1* (cyclin D1) and *CCNE1* (cyclin E1) promoters. However, the depletion of ARID2 increased E2F1 binding to the *CCND1* and *CCNE1* gene promoters, which correlated with an increase in RNA polymerase II (RNA Pol II) association and histone acetylation at these promoters (Figure 5, Supplementary Figures 4 and 5). ARID2 did not affect the binding of E2F1 and RNA PII to the cyclin A1 promoter region (Supplementary Figure 6).

In order to further examine the direct interaction between E2F1 and ARID2 proteins, we performed co-immunoprecipitation (Co-IP) assays in SMMC-7721 cells. Data indicated that endogenous E2F1 protein was co-immunoprecipitated with antibodies against ARID2 (Figure 5E). The subcellular localization of E2F1 and ARID2 was confirmed by confocal immunofluorescence staining. Results indicated nuclear co-localization of ARID2 and E2F1 proteins (Figure 5F). These data, in line with the ChIP results, suggest that ARID2 physically interacts with E2F1 protein and dissociates activator E2F1 at the *CCND1* and *CCNE1* promoters, resulting in suppression of cyclin D1 and cyclin E1.

### Dissociation of E2F1 is responsible for ARID2-induced cyclin D1 and cyclin E1 repression

**Figure 6:**
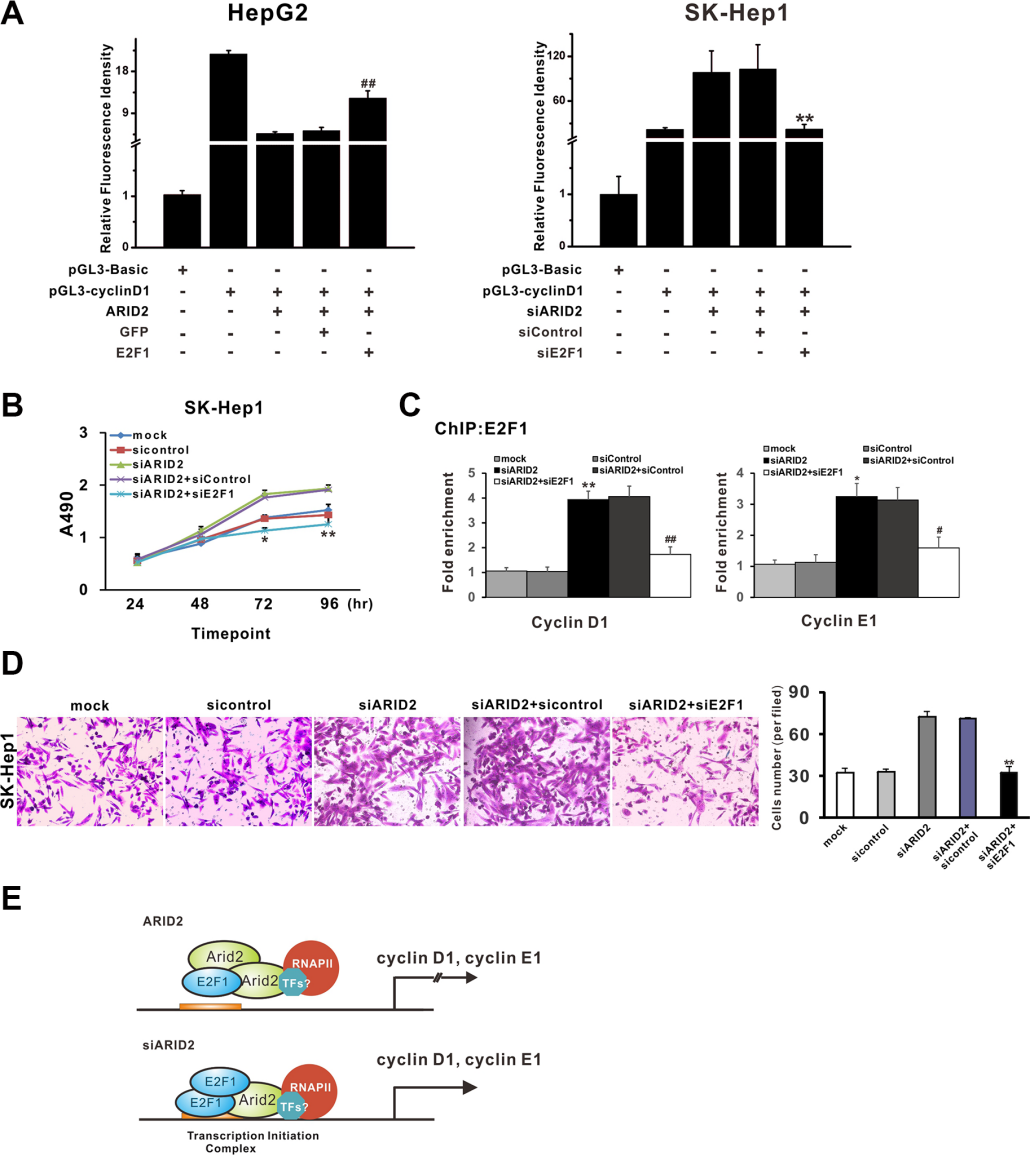
Dissociation of E2F1 is responsible for ARID2-mediated repression of cyclin D1 and cyclin E1 (**A**) Luciferase assay of human *CCND1* and *CCNE1* promoter constructs in HepG2 cells or SK-Hep1 cells. HepG2 cells or SK-Hep1 cells were transfected with pGL3-cyclin D1 or pGL3-Basic control. At 24 hours after transfection, HepG2 cells were infected with Ad-ARID2 + Ad-E2F1, or Ad-ARID2 + Ad-GFP. SK-Hep1 cells were infected with AdR-siARID2 + AdR-siE2F1 or AdR-siARID2 + AdR-siControl. At 36 hours after infection, cells were collected for luciferase assays. Results are presented as the mean relative luciferase activity against the activity of the pGL3-Basic control sample ± SD of three independent experiments; **p < 0.01 (siARID2 + siE2F1 vs. siARID2 + siControl); ^##^*p* < 0.01 (ARID2 + E2F1 vs. ARID2 + GFP). (**B**) Cell proliferation curves: SK-Hep1 cells were infected with AdR-siARID2, AdR-siARID2 + AdR-siE2F1, AdR-siARID2 + AdR-siControl, or AdR-siControl. At 12 hours after infection, cells were subjected to MTS assay. Data are presented as means ± S.D. *p < 0.05, **p < 0.01 (siARID2 + siE2F1 vs. siARID2 + siControl). (**C**) ChIP-qPCR analysis. Data represent the means of three independent experiments ± SD; **p < 0.01 (siARID2 vs. siControl); ^##^*p* < 0.01 (siARID2 + siE2F1 vs. siARID2 + siControl). (**D**) Transwell assay of cell migration in SK-Hep1 cells. Cells were treated as described in Figure 6B. Data represent the results of three independent experiments ± SD; **p < 0.01(siARID2 + siE2F1 vs. siARID2 + siControl), magnification: 200 ×. (**E**) A schematic model of ARID2-mediated repression of cyclin D1 and cyclin E1. Data indicate that ARID2 physically interacts with E2F1and induces dissociation of activator E2F1 and RNA Pol II as well as histone deacetylation, contributing to the transcriptional repression of cyclin D1 and cyclin E1. However, *ARID2* knockdown enhances binding of activator E2F1 and induces active histone modification, thus promoting cyclin D1 and cyclin E1 transcription.

In order to explore the possible roles of E2F1 in ARID2-induced suppression of cell proliferation and migration, we evaluated cell growth and invasion in *ARID2*-expressing HepG2 cells that ectopically expressed E2F1 protein (ARID2 + E2F1) or in SK-Hep1 cells in which both ARID2 and E2F1 were depleted (siARID2 + siE2F1). Overexpression of E2F1 partially reversed ARID2-induced repression of cyclin D1 promoter activity, whereas knockdown of E2F1 decreased cyclin D1 transcriptional activity in ARID2-depleted SMMC-7721 cells (Figure 6A). Furthermore, we found that overexpression of E2F1 efficiently reversed ARID2-induced repression of cell proliferation and migration in HepG2 cells (data not shown). Meanwhile, E2F1 silencing inhibited tumor cell growth and invasiveness, accompanied by markedly decreased binding of E2F1 at *CCND1* and *CCNE1* gene promoters in ARID2-depleted cells (Figure 6B–6D). These data confirmed that dissociation of E2F1 is responsible for ARID2-mediated repression of cyclin D1 and cyclin E1 repression.

## DISCUSSION

Recent exome sequencing studies of hepatocellular carcinoma indicate that inactivating mutations in SWI/SNF subunits are involved in the tumorigenesis of HCC [16, 17]. As ARID2 has only recently been implicated as a tumor suppressor in hepatocarcinogenesis, relatively few studies have evaluated *ARID2* expression in tumor tissues or explored the mechanism by which ARID2 affects downstream protein expression and function.

In the present study, we show that *ARID2* expression is significantly downregulated in HCC tissues. The suppression of *ARID2* expression in hepatoma cells facilitated G1/S transition associated with transcriptional upregulation of E2F1, cyclin D1, and cyclin E1, induction of CDK4, and phosphorylation of Rb. Furthermore, our findings indicate that ARID2 induces dissociation of E2F1 and RNA Pol II at regions upstream of the transcription start sites of cyclin D1 and cyclin E1. Moreover, we found that the expression levels of these genes were substantially upregulated following knockdown of *ARID2*, supporting cyclin D1 and cyclin E1 as direct targets of ARID2. Our results reveal a tumor suppressor function of ARID2, which involves targeting the Rb-E2F signaling pathway and thus regulating E2F-downstream genes.

Although the tumor suppressor functions of SWI/SNF subunits have been verified in a variety of tumors, the mechanisms underlying the crosstalk of SWI/SNF factors with oncogenic pathways or transcriptional factors in the modulation of cellular behavior, such as cell proliferation and differentiation, remain poorly understood. Several SWI/SNF-multicomplexed factors have been reported to interact with transcriptional factors, such as Myc [18] and Nanog [19], to modulate or maintain chromatin compaction, thereby inducing senescence associated with target gene suppression. The present study demonstrates that physical interactions between ARID2 and the transcriptional factor E2F1 play pivotal roles in ARID2-mediated suppression of cyclin D1 and cyclin E1, supporting the notion that *ARID2* acts as a tumor suppressor by targeting the Rb-E2F signaling pathway.

Several studies have shown that the Rb/E2F family proteins interact with various chromatin-modifying complexes, such as CHD8, RBL2/p130, L3MBTL1, BRG1, and mSin3B, involved in maintaining suppressive chromatin structure and stable transcriptional repression [20–23]. The presence of E2F-binding sites has been demonstrated in multiple G1/S-phase genes such as those encoding cyclin B1 and cdc2 [24]. Our findings show that the chromatin-remodeling protein ARID2 induces dissociation of the activator E2F1, AcH3, and RNA Pol II from the target promoter, thus contributing to the repression of cyclin D1 or cyclin E1 (Figure 6E).

Additionally, we found that *ARID2* knockdown promotes HCC cell migration and invasion, whereas overexpression of *ARID2* inhibits hepatoma cell migration. Previous studies have demonstrated that decreased expression of *ARID1A,* which encodes another protein of the ARID family, enhances gastric cancer cell or HCC cell migration via transcriptional silencing of E-cadherin [25, 26]. Moreover, the BRG-1 subunit of the SWI/SNF complex has been reported to regulate CD44 expression and play a critical role in regulating cellular adhesion and metastasis [27, 28]. Further investigation is required to determine whether ARID2 affects the expression of markers and regulators of epithelial-mesenchymal transition (EMT).

In conclusion, the present study reveals that ARID2 negatively regulates cell-cycle progression and cellular proliferation, and represses cyclin D1 and cyclin E1 expression by targeting the Rb-E2F signaling pathway, implying that this tumor suppressor functions as a “gatekeeper.” Further studies are required to investigate whether additional SWI/SNF subunits or transcriptional regulators are implicated in ARID2-mediated gene repression. Elucidation of the mechanisms by which the tumor suppressor gene *ARID2* plays a role in cancer pathogenesis should pave the way for the development of novel diagnostic and therapeutic strategies against HCC.

## MATERIALS AND METHODS

### Patient samples

HCC tissues and paired adjacent non-tumorous tissues were obtained from 40 patients who underwent surgery for HCC at the 2nd Affiliated Hospital of Chongqing Medical University between 2014 and 2015. Patients had not received chemotherapy or radiation therapy before surgery. This study received the approval of the Institutional Ethical Review Board of Chongqing Medical University and informed consent was obtained from participating patients. All liver specimens were collected immediately after surgery and stored at −80°C until further use.

### Cell cultures

The human HCC cell line HepG2 was obtained from the American Type Culture Collection (ATCC, VA, USA). SK-Hep1 and SMMC-7721 cells were obtained from the Cell Bank of the Chinese Academy of Sciences. Normal human liver cells (LO2) were acquired from the Chinese Academy of Sciences (Shanghai, China). MIHA cells (immortalized liver cell line) were kindly provided by Dr. Ben. C.B. Ko (The Hong Kong Polytechnic University) [29]. Cells were cultured in DMEM (Hyclone) supplemented with 10% fetal bovine serum, 100 unit/mL of penicillin, and 100 mg/mL of streptomycin at 37°C in 5% CO_2_. HepG2 cells were cultured in Minimum Essential medium (MEM, Hyclone).

### Adenoviruses and reporter plasmids

Three pairs of oligonucleotides containing siRNA (Supplementary Table 2) target sites for the coding regions of *ARID2* and *E2F1* were designed and subcloned into the SfiI site of the pSES1 vector (from Dr. T-C He, University of Chicago, USA) to generate adenoviruses AdR-siARID2 and AdR-siE2F1, using the AdEasy system.

The full-length cDNA of *ARID2* (coding sequence of NM_152641) was subcloned from plasmid pEZ-M02-ARID2 (GeneCopoeia plasmid EX-E2538-M02-5) into the shuttle vector pAdTrack-TO4 (from Dr. T-C He). The adenoviral recombinant pAd-ARID2 was also generated using the AdEasy system [30]. An analogous adenovirus expressing only GFP (Ad-GFP) and a scrambled shRNA (AdR-siControl) expressing RFP were used as controls.

Wild-type (WT) Cyclin D1 (*CCND1*) and Cyclin E1 (*CCNE1*) promoter-luciferase reporters were generated by cloning ∼1-kb and ∼400-bp PCR fragments into the pGL3-Basic vector (Promega, Madison, WI, USA; #E1751), respectively.

### Dural-luciferase assay

Cells were plated in 24-well plates and transfected with 0.5 μg each of *CCND1* or *CCNE1* promoter construct along with 100 ng/well of pRL-TK (an internal control) using Lipofectamine^TM^ 2000 (Invitrogen, Carlsbad, CA, USA). At 16 h after transfection, cells were infected with Ad-ARID2 or Ad-GFP. At 24 h after infection, cells were lysed and subjected to dual-luciferase reporter assay using a Dual-Luciferase Assay Kit (Promega, Madison, WI, USA) following the manufacturer's protocol. Assays were performed in triplicate and results expressed as means ± standard deviation (SD).

### Tumor transplantation in animal models

For the subcutaneous (s.c.) xenograft tumor model, total number of 25 nude mice (4–6 weeks old, male, 20–25 g) was randomly divided into five groups. SMMC-7721 cells were mock-infected or infected with Ad-GFP, Ad-ARID2, AdR-siARID2, or AdR-sicontrol for 16 hours and collected for subcutaneous injection (1 × 10^6^ cells/injection) into the flanks of athymic nude. Tumor volume (V) was monitored by measuring the length (L) and width (W) using calipers at 7-day intervals, for consecutive 7 weeks, as follows: V[cm^3^] = (length[cm]) × (width[cm] × (width[cm])/2. After 7 weeks, the mice were sacrificed and tumor tissues were removed for histological analysis.

For the orthotopic implantation model, SMMC-7721 cells were co-infected with AdR-FLuc and AdR-siARID2, AdR-sicontrol, Ad-GFP, or Ad-ARID2. After 24 hours, the infected cells (1 × 10^5^ cells/injection) were collected and implanted into the left lobes of the livers of nude mice (5 animals in each group).

*In vivo* tumor formation was monitored by bioluminescent imaging (BLI) using a Xenogen IVIS 200 imaging system at week 7 after implantation. The resulting images were automatically superimposed using the IVIS Living Image (Xenogen, Alameda, CA, USA)) software to facilitate matching the observed luciferase signal with its corresponding location in the mouse. At 7 weeks after implantation, mice were sacrificed and liver tissues were retrieved for histological examination.

The use and care of animals was approved by the Institutional Animal Care and Use Committee at Chongqing Medical University.

### RNA isolation and Real-Time PCR

Cells were mock-infected or infected with Ad-GFP, Ad-ARID2, AdR-siARID2, or AdR-sicontrol. Thirty-six hours after infection, total RNA was isolated using TRIzol (Invitrogen, Rockville, MD) following the manufacturer's instructions, as previously described [31]. RNA was reverse-transcribed using Moloney murine leukemia virus reverse transcriptase (Promega) in the presence of random hexamers (Promega). PCR-based amplification of the respective genes was carried out using the cDNA products as templates. SYBR Green-based qPCR analysis was carried out using the DNA Engine Opticon 2 real-time PCR detection system (Bio-Rad, CA, USA). Relative expression was calculated as a ratio of the expression of the specific transcript to that of glyceraldehyde 3-phosphate dehydrogenase (GAPDH). Each sample was analyzed in triplicate. The primer sequences used for PCR are listed in Supplementary Table 2.

### Immunohistochemistry

Liver tissue samples were fixed in 4% paraformaldehyde and embedded in paraffin according to standard procedures. Sections were incubated with the following primary antibodies: anti-ARID2, anti-E2F1, anti-Ki-67, anti-cyclin D1, and anti-cyclin E1 (described above). Subsequently, the slides were incubated with Envision System-HRP and visualized using DAB substrate (Maixin-Bio, Fuzhou, China). IHC scoring for ARID2 was performed by two independent pathologists. The IHC scores of nuclear immunoreactivity to ARID2 were assigned as follows: 0 (< 10% positive cells); 1 (10%–30%); 2 (30%–50%); 3 (> 50%). Staining was analyzed according to the percentage of positively stained cells and staining intensity using Image-Pro plus 6.0 software (Media Cybernetics, Inc., Bethesda, MD).

### Western blot analysis

Whole protein lysates of cells or liver tissues were collected using cell lysis buffer (Beyotime, Shanghai, China) with a complete cocktail of protease inhibitors (Roche, Mannheim, Germany). Protein concentrations were determined by a BCA protein assay (Thermo, Waltham, MA, USA). Protein samples were separated by 10% SDS/PAGE and electrotransferred to PVDF membranes (Millipore, Billerica, MA, USA). The blots were probed with the indicated antibodies. Secondary antibodies coupled to horse-radish peroxidase were purchased from Abcam. Proteins bands were visualized using Super Signal West Pico Chemiluminescent substrate Kits (Millipore). Western blots were quantitated by densitometric analysis using the ImageJ software program.

### MTS proliferation assay

Cells were plated in 96-well plates at 2 × 10^3^ cells per well and cultured for 24, 48, 72, and 96 hours. The absorbance at 490 nm was measured after incubation with 20 μL of MTS labeling reagent solution (Promega) for 2 hours.

### Cell-cycle analysis

Cells were harvested and subjected to cell cycle analysis, as previously described [32]. Samples were processed using a FACS Calibur machine (BD Biosciences, Franklin Lakes, NJ, USA). The acquired data were analyzed using CellQuest software.

### Cell migration assay

Cells infected with AdR-siControl, AdR-siARID2, Ad-GFP, or Ad-ARID2 were suspended in 200 μL of serum-free DMEM medium, at a concentration of 1 × 10^5^ cells/well, and then placed in the upper chamber of the Transwell. DMEM medium plus 10% fetal bovine serum was placed in the bottom well and cells were allowed to migrate for 10 h at 37°C. Cells were fixed in methanol and stained with crystal violet. The number of migrated cells were determined by counting five fields (200 ×) on each membrane and calculated as the mean number of cells per field.

### Immunofluorescence

Cells were fixed with 4% paraformaldehyde and permeabilized using 0.3% Triton X-100. After incubation with blocking solution containing 10% normal goat serum, cells were labeled with appropriate primary antibodies diluted in PBS with 1% BSA and incubated with Alexa Fluor 488 or 594 secondary antibody (Invitrogen) in PBS. Cells were counterstained with DAPI for 10 min to label nuclei. The presence of ARID2 or E2F1 was visualized under a laser scanning confocal microscope (Nikon A1R, Tokyo, Japan).

### Co-IP assay

SMMC-7721 cells were resuspended in 1 mL of RIPA lysis buffer (Beyotime) and pre-cleared with protein G agarose beads (Millipore, Billerica, MA, USA) for 1 hour. Supernatants were incubated with ARID2 or E2F1 antibodies overnight at 4°C, followed by a two-hour incubation with protein G agarose beads. Immune complexes were resolved by 10–15% SDS/PAGE and transferred to PVDF membranes, then subjected to immunoblot analysis with the indicated antibodies.

### ChIP analysis

ChIP was carried out as described previously [33]. Briefly, sonicated cell lysates were subjected to immunoprecipitation using 5 μg of the respective primary antibody (normal IgG served as control). Following elution, DNA was extracted and subjected to PCR analysis. Two pairs of primers specific for the human *CCND1*, *CCNE1*, and *CCNA1* promoters were used for PCR (Supplementary Table 2). For ChIP-qPCR, the immunoprecipitated DNA was quantitated by real-time PCR. The enrichment of epigenetic modification marks at the examined regions was quantitated relative to the input control.

### Statistical analysis

All values were presented as means ± standard deviation (SD). Statistical significance was determined using one-way ANOVA for multiple comparisons and Student's *t*-test was used to compare two groups. Correlations between ARID2 protein expression and individual clinicopathologic parameters were evaluated using a nonparametric χ^2^ test. Probability values (p) of < 0.05 were considered to represent statistical significance.

## Supplementary Materials


