# Supplementary material for: Chromatin remodeling gene ARID2 targets cyclin D1 and cyclin E1 to suppress hepatoma cell progression
Source: Oncotarget. 2016 Jun 23;7(29):45863–75. doi: 10.18632/oncotarget.10244 (PMC5216766; doi:10.18632/oncotarget.10244)
Supplement: Supplementary file 1 [file oncotarget-07-45863-s001.pdf]

Chromatin remodeling gene *ARID2* targets cyclin D1 and cyclin E1 to suppress hepatoma cell progression

Supplementary Materials

MATERIALS AND METHODS

Antibodies

The following primary antibodies were used in this study: polyclonal rabbit anti-ARID2 antibody (Abcam, Cambridge, UK; ab113283), polyclonal rabbit anti-CDK2 antibody (Bioworld, Minneapolis, MN, USA; BS1050), monoclonal mouse anti-CDK4 antibody (Bioworld, MB0027), polyclonal rabbit anti-p16 antibody (Bioworld, BS1265), polyclonal rabbit anti-p27 antibody (Bioworld, BS3714), polyclonal rabbit anti-E2F1 antibody (Santa Cruz, CA, USA; sc-193), polyclonal rabbit anti-cyclin D1 antibody (Cell Signaling Technology, #2978),polyclonal rabbit anti-cyclin E1 antibody (Bioworld, BS1085), polyclonal rabbit anti-cyclin A1 antibody (Bioworld, BS1083), monoclonal mouse anti-Rb antibody (Santa Cruz, sc-74562), monoclonal mouse anti-p-Rb antibody (Santa Cruz, sc-135776), polyclonal rabbit anti-c-myc antibody (Santa Cruz, sc-764), polyclonal rabbit anti-Ki67 antibody (Bioworld, BS1454), and monoclonal mouse anti-GAPDH antibody (Beyotime Biotechnology, Jiangsu, China; #AG019-1).

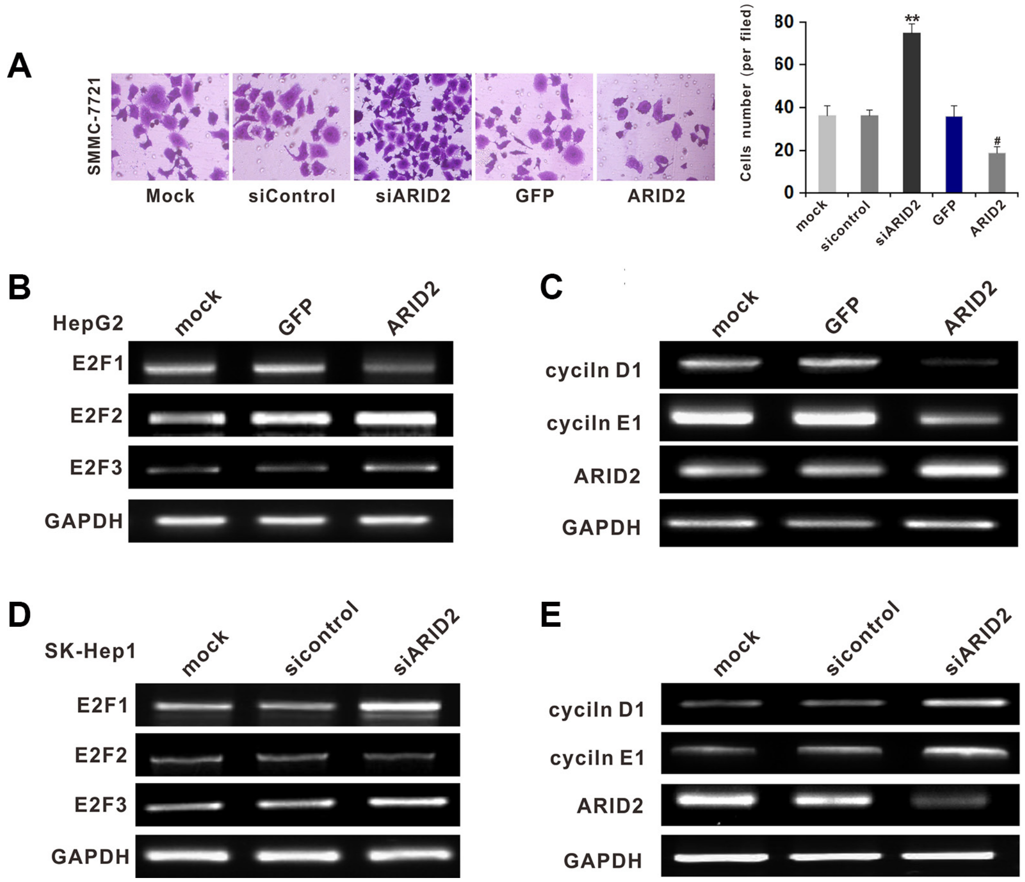

Supplementary Figure S1: ARID2 participates in the regulation of the Rb-E2F signaling pathway. (**A**) Transwell assay of cell migration in SMMC-7721 cells. Cells were infected with Ad-ARID2, Ad-GFP, AdR-siARID2, or AdR-siControl. Data represents the results of three independent experiments ± SD. $^{**}p$AdR-siARID2/siControl < 0.01, $^{\#}p$Ad-ARID2/Ad-GFP < 0.05. Magnification: 200×. (**B–E**) HepG2 cells were infected with adenoviruses expressing ARID2 (Ad-ARID2) or vector control (Ad-GFP) (**B** and **C**). SK-Hep1 cells were infected with AdR-siARID2 or siRNA control (AdR-siControl) (**D** and **E**). At 36 hours after infection, the mRNA levels of downstream molecules such as the E2F family members (E2F1-3), cyclin D1, and cyclin E1 were determined by RT-PCR.

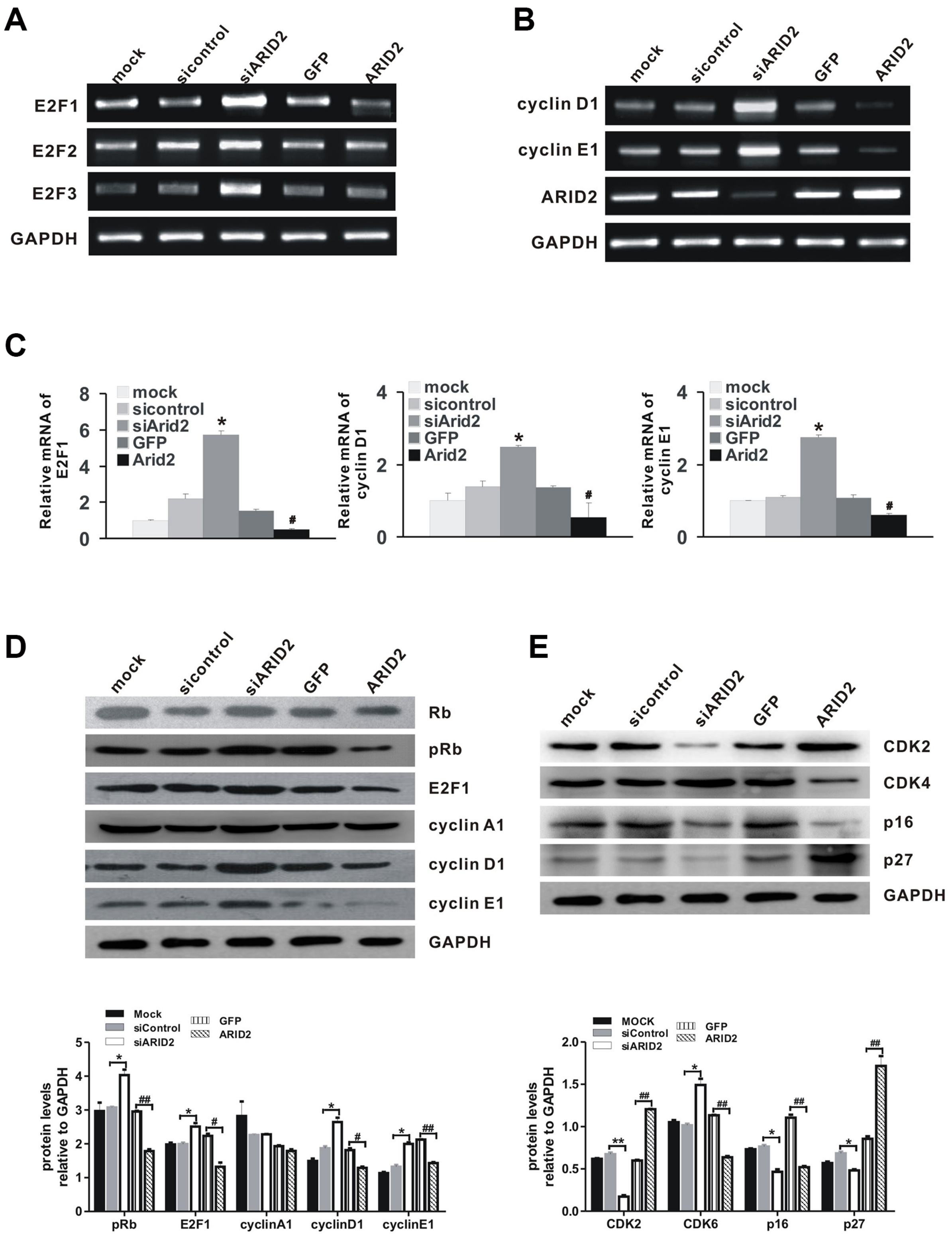

Supplementary Figure S2: ARID2 regulates molecules downstream of the Rb-E2F signaling pathway in SMMC-7721 cells. Cells were treated as described in Supplementary Figure 1A. (**A**) The mRNA levels of E2F family members (E2F1-3) were determined by RT-PCR. (**B**) The relative mRNA levels of cyclin D1 and cyclin E1 were measured by RT-PCR. (**C**) RT-PCR analysis of E2F1, cyclin D1, and cyclin E1. Data represents the three independent experiments ± SD; *pAdR-siARID2/siControl < 0.05, $^{\#}p$Ad-ARID2/Ad-GFP < 0.05. (**D**) and (**E**) The protein levels of selected Rb-E2F-dependent genes, including Rb, phosphorylated-Rb (p-Rb), E2F1, cyclin D1, cyclin E, cyclin A (D), and cell-cycle regulators CDK2, CDK4, p16, and p27 (E) were determined by western blot. Integrated density of proteins were quantitatively analyzed using ImageJ software. *$p < 0.05$, **$p < 0.01$(siARID2 vs. siControl); $^{\#}p < 0.05$, $^{\#\#}p < 0.01$(ARID2 vs. GFP).

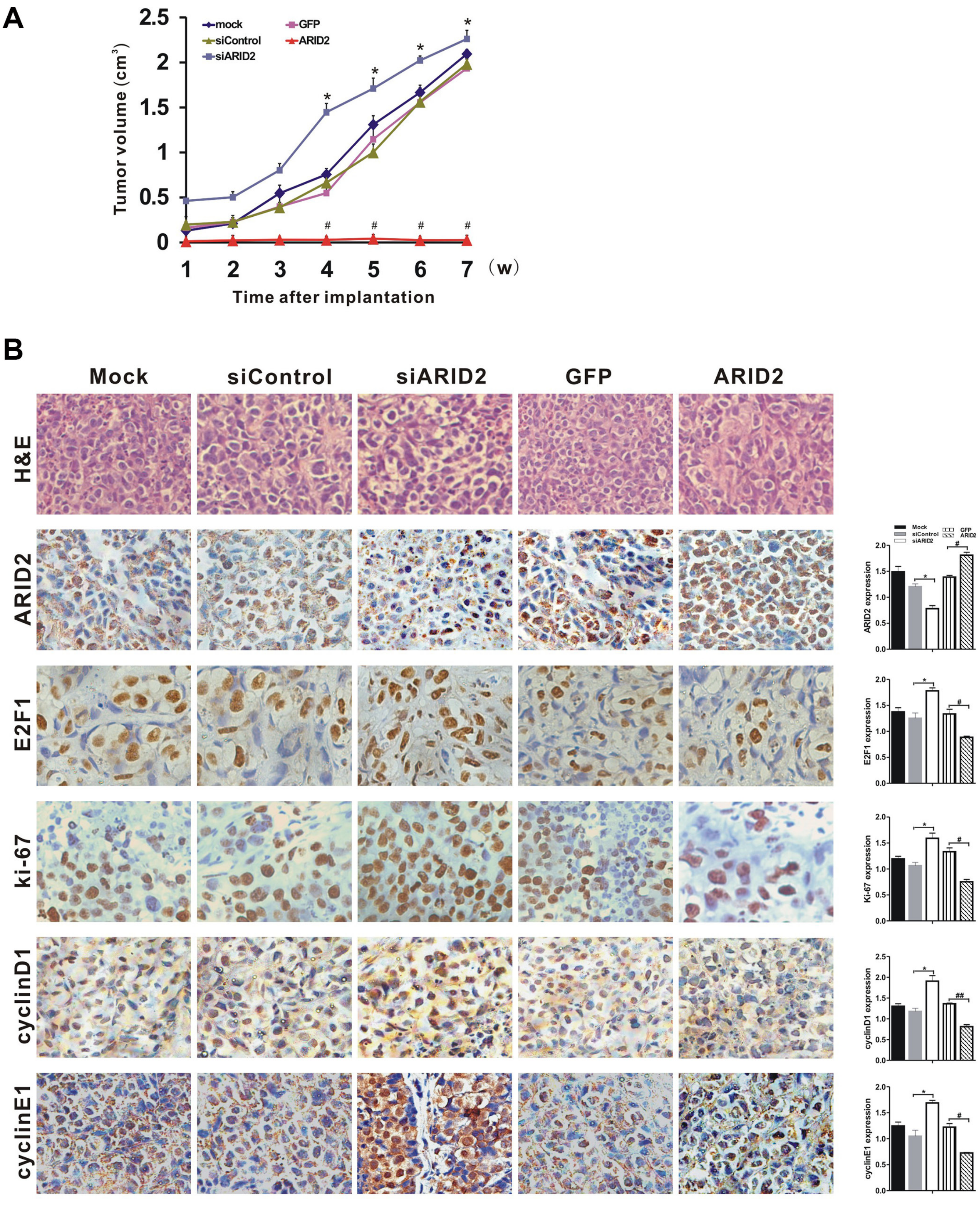

Supplementary Figure S3: ARID2 inhibits tumor growth in xenograft model. SMMC-7721 cells were infected with AdR-siARID2, AdR-siControl, Ad-ARID2, or Ad-GFP control for 15 hours. Then, the infected cells were injected subcutaneously into the flanks of athymic mice (5 mice per group). At 7 weeks after implantation, animals were sacrificed and tumor masses were retrieved. (**A**) Analysis of tumor volume over a 7-week time course. Data represent the means ± SD; *pAdR-siARID2/siControl < 0.05, $^{\#}p$Ad-ARID2/Ad-GFP < 0.05 (**B**) Immunohistochemical staining of ARID2, E2F1, ki-67, cyclin D1, and cyclin E1 in tumor tissues. Magnification: 400 ×. The staining intensity was assessed using Image-Pro 6.0 software. *$p < 0.05$, **$p < 0.01$(siARID2 vs. siControl); $^{\#}p < 0.05$, $^{\#\#}p < 0.01$(ARID2 vs. GFP).

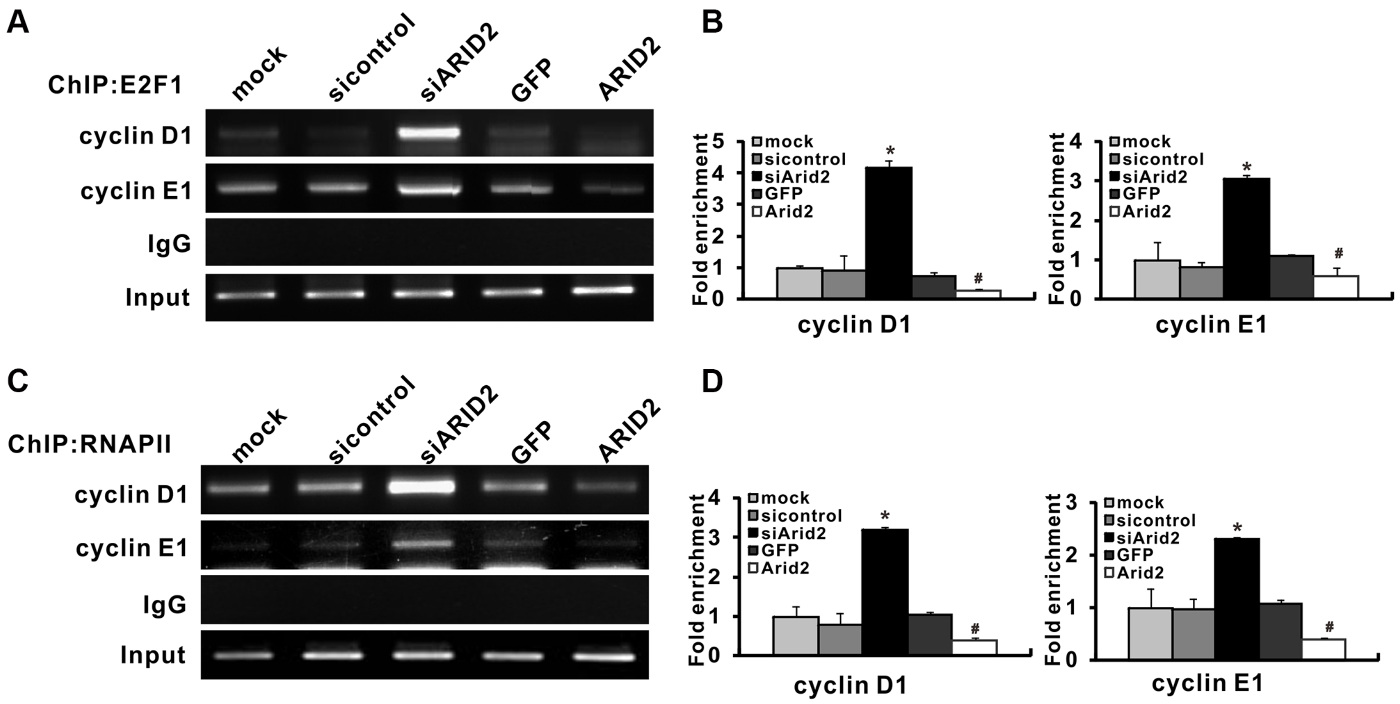

Supplementary Figure S4: ARID2 represses E2F1 binding to *CCND1* and *CCNE1* promoters in SMMC-7721 cells. SMMC-7721 cells were treated for 24 hours as described in Supplementary Figure 3. Then, cell lysates were analyzed by ChIP of the *CCND1* (cyclin D1) and *CCNE1* (cyclin E1) promoters using antibodies against E2F1 (**A**) and RNAPII (**C**). IgG served as negative control. Data represent the results of three independent experiments (**B** and **D**). Error bars represent ± SD values. **p*AdR-siARID2/siControl < 0.05, [#]*p*Ad-ARID2/Ad-GFP < 0.05.

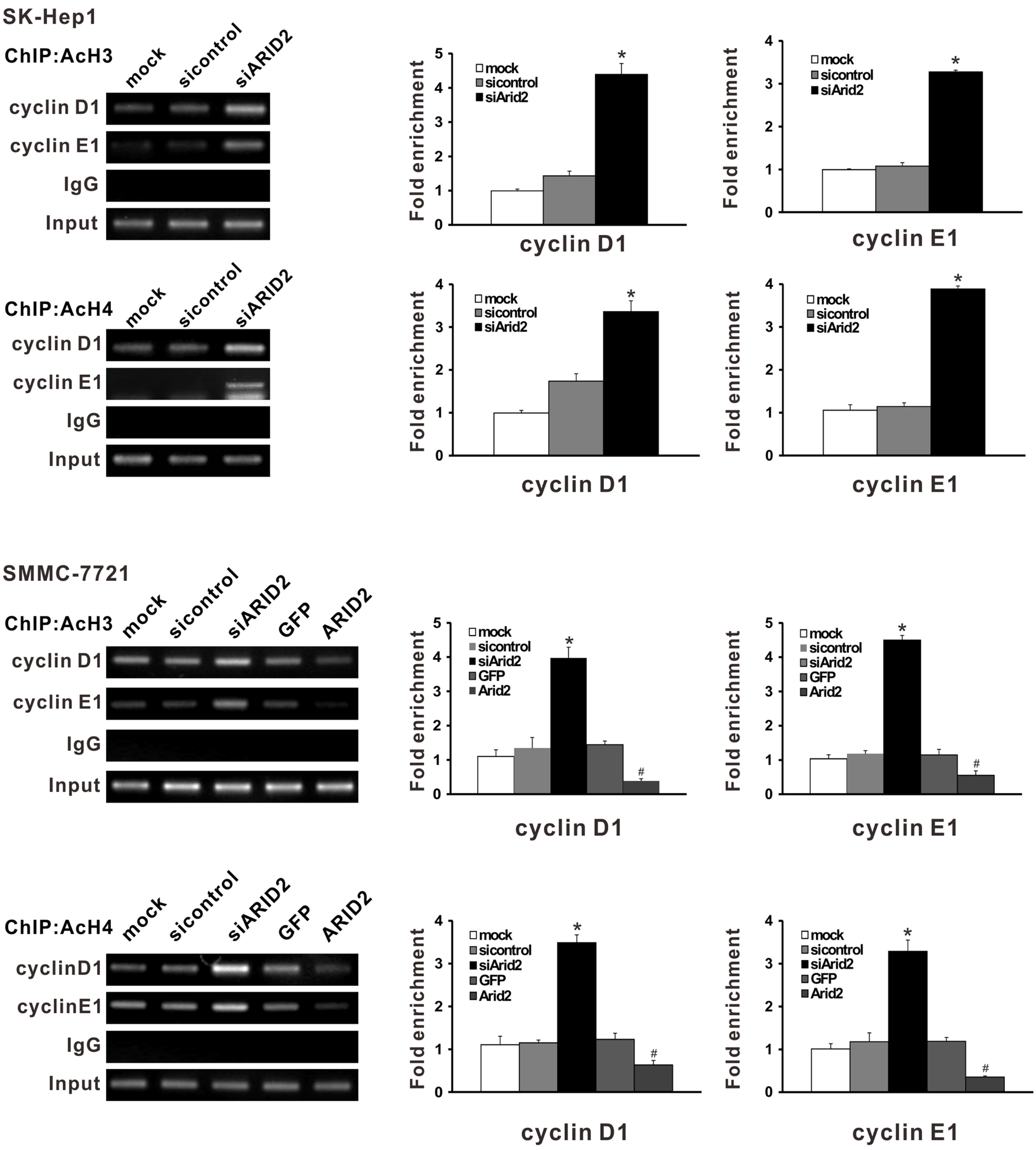

Supplementary Figure S5:ARID2 induces deacetylation of CCND1 and CCNE1 promoters in SK-Hep1 and SMMC-7721 cells. SK-Hep1 cells were mock-infected or infected with AdR-siARID2 or AdR-siControl for 24 hours. SMMC-7721 cells were treated as described in Supplementary Figure 4. Then, cell lysates were analyzed by ChIP using antibodies against AcH3 and AcH4. IgG served as negative control. Data represent the results of 3 independent experiments ± SD. *pAdR-siARID2/siControl < 0.05, $^{\#}p$Ad-ARID2/Ad-GFP < 0.05.

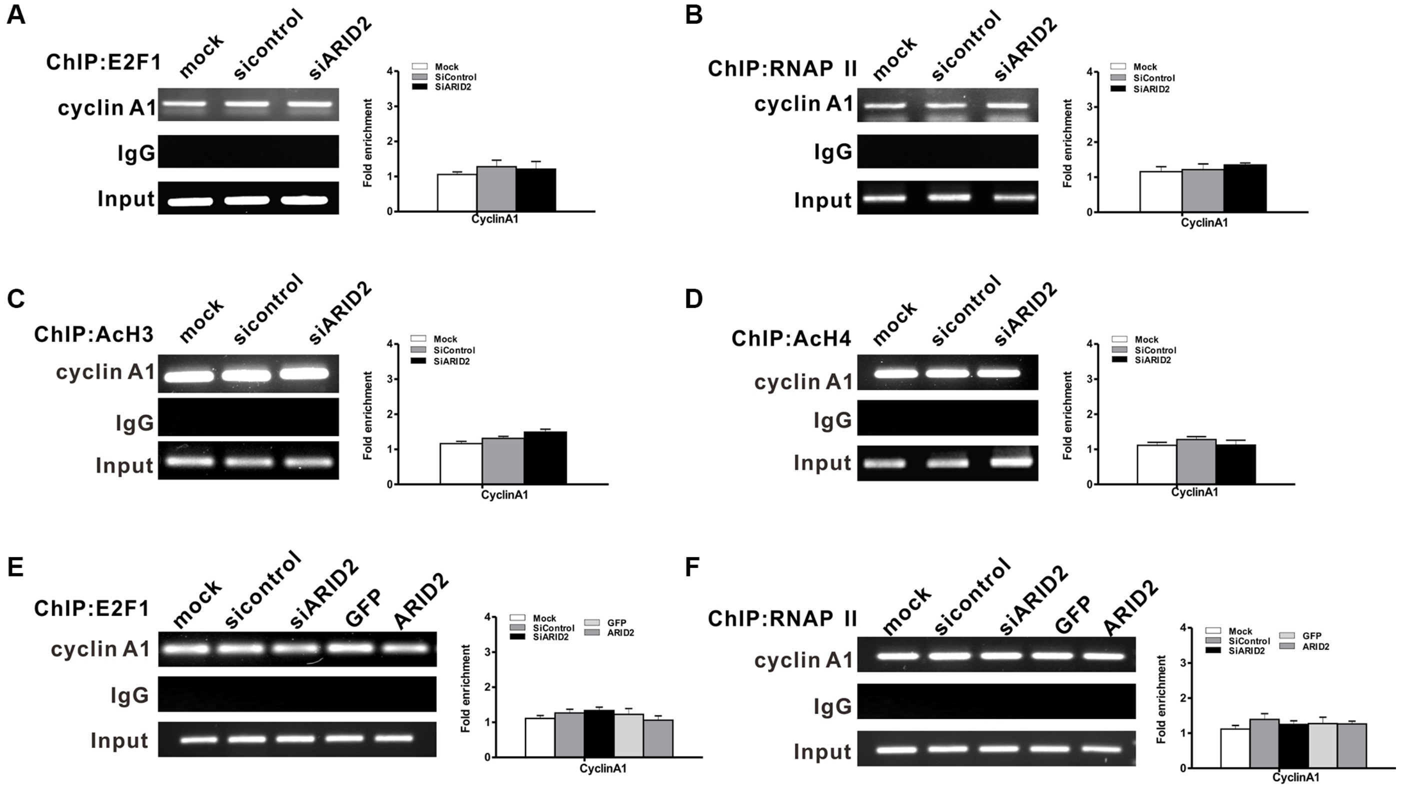

Supplementary Figure S6: ARID2 has no effect on the binding of E2F1/RNA Pol II to the *Cyclin A1* promoter in SK-Hep1 and SMMC-7721 cells. SK-Hep1 cells and SMMC-7721 cells were treated as described in Supplementary Figure 5. Then, cell lysates were analyzed by ChIP using antibodies against E2F1, RNA Pol II, AcH3, and AcH4. IgG served as the negative control. Data represent the results of three independent experiments ± SD.

Supplementary Table S1: Clinical correlation between levels of ARID2 protein expression and clinicopathologic parameters of HCC patients (n = 40)

	Frequency(%)	ARID2 expression (Tumor/peritumoral)		P Value
		Low(-)	High(+)	
Sex				
Male	37(92.5)	24	13	0.545
Female	3(7.5)	1	2	
Age				
≤ 55	29(72.5)	22	7	0.99
> 55	11(27.5)	9	2	
Tumor size, cm				
> 5	30(75)	20	10	0.159
≤ 5	10(25)	4	6	
Alpha fetoprotein, ng/mL				
≤ 100	16(40)	10	6	0.95
> 100	24(60)	15	9	
Hepatitis B surface antigen				
Positive	32(80)	24	8	0.96
Negative	8(20)	6	2	
Cirrhosis				
Negative	24(60)	20	4	0.690
Positive	16(40)	12	4	
Histological differentiation				
Well	7(17.5)	3	4	0.005*
Moderate/Poor	33(82.5)	31	2	
Capsular invasion				
Absent	17(42.5)	13	4	0.98
Present	23(57.5)	17	6	
Intrahepatic metastasis				
Absent	4(10)	3	1	0.96
Present	36(90)	29	7	
Distant metastasis				
Absent	12(30)	5	7	0.166
Present	28(70)	19	9	
Recurrence				
Absent	18(45)	13	5	0.430
Present	22(55)	19	3	

A total of 40 HCC tissues with defined clinical data were chosen for this correlation study. High tumoral ARID2 expression was considered > 1.5-fold increases relative to the peritumoral liver tissue. *P < 0.05.

Supplementary Table S2: Primer sequences

	Sense Primer (5′–3′)	Antisense Primer (5′–3′)
**siArid2 primers**		
siARID2 1	A TGT AGG AAA TGG TGA GAT A TTTT	AT ATC TCA CCA TTT CCT ACA TTTT
siARID2 2	A TTT CCC AGA TGC TCC ATT C TTTT	AG AAT GGA GCA TCT GGG AAA TTTT
siARID2 3	A CCA CAA GGG ACT TTA GAT A TTTT	AT ATC TAA AGT CCC TTG TGG TTTT
**siE2F1 primers**		
siE2F1 1	A GGA AAG TGA GGG AGG GAG A TTTT	AT CTC CCT CCC TCA CTT TCC TTTT
siE2F1 2	A CCT CAT AAC TCG AAG AAA G TTTT	AC TTT CTT CGA GTT ATG AGG TTTT
siE2F1 3	A CCA AGA AGT CCA AGA ACC A TTTT	AT GGT TCT TGG ACT TCT TGG TTTT
**ARID2 CDS primer**	ATA GC GGCC GC ACC atg gcaaactcgacggggaaggcgc	GCG TCTAGA TCA CTGCA GCATTTCTGA GTCTT
**Real-time PCR primers**		
E2F1	ATGTTTTCCTGTGCCCTGAG	ATCTGTGGTGAGGGATGAGG
E2F2	GGCCAAGAACAACATCCAGT	TGTCCTCAGTCAGGTGCTTG
E2F3	TGAACAAGGCAGCAGAAGTG	TTTGACAGGCCTTGACACTG
cyclin D1	AGTGCGTGCAGAAGGAGATT	CACAACTTCTCGGCAGTCAA
cyclin E1	TGGCGTTTAAGTCCCCTGAC	AAGGCCGAAGCAGCAAGTAT
cyclin A1	ACCCCAAGAGTGGAGTTGTG	GGAAGGCATTTTCTGATCCA
ARID2	GATTGTAAGCCAGCCAGCTC	CATGGCAGTAGGGACTTGGT
GAPDH	CGACCACTTTGTCAAGCTCA	AGGGGTCTACATGGCAACTG
**pGL3-cyclinD1**	TAC GGTACC GCAAATTCTAAAGGTGAAGGGACGTCT	CAT AAGCTT GAGGCTCCAGGACTTTGCAACTTCAAC
**pGL3-cyclinE1**	TAC GGTACC CCCCACACATCCCCTTGGCTCAGCCCT	CAT AAGCTT GACATTTAAAATCCCTGCGCGCGGAAC
**ChIP primers**		
cyclin D1		
Primer 1 (–1861–1691 nt)	AGCCTCTTTATGCCCTGCTG	TCATAAAATCCCGACCCGGC
Primer 2 (–721–547 nt)	AAGGAAATGCTGGCCACCAT	TGTGCAAGTTTCATTCCGGC
cyclin E1		
Primer 1 (–1367–1097 nt)	CACTATGCCAAGAACTGACA	TGTTGCTCAGGCTGGTCTCA
Primer 2(–325–88 nt)	GTGTTTACATTCCACCCGCG	GCAGGGACGGGGAATCAG
cyclin A1	GACGGCTGAAAGTCACTGGA	TGTTCGTGGAACCTGTCGTT
